# Control Work

**Published:** 1900-04

**Authors:** 


### CONTROL WORK.

UNDER date of April 1, 1900, the Department of Agriculture of North Carolina issued under advice of the State Veterinarian, Dr. Cooper Curtice, the following bulletin :

Acclimation disease in bulls has for years been a bar against improvement by introducing thoroughbred stock.

By observing a few precautions bulls may be brought to this State to any portion of the " stock law " district at any time of year without danger of loss.

If there are no cattle-ticks on the cattle of the farm, either transport the imported bull in a wagon from the depot (not unloading into the cattle pen), or drive him in the middle of the road after greasing his legs, and do not allow him to touch grass by the way. Grease them again when they arrive. Or on infested farms prepare a yard and shed in a field which has been cultivated during two years and no cattle allowed thereon. Build a double fence, the inside being of high, tight boards, and the outer, at a few feet distant, of wire that will keep stock away. Provide shade.

Set aside a special pen in which cows may be served. Clean each cow of all ticks before admitting her, and thoroughly grease her. When served take both away from this pen.

Feed correctly and allow plenty of exercise.

A certificate stating that the bulls are not infected with any communicable disease, and signed by the authorities of the State they started from, should accompany each importation. Buy any breed of cattle where quarantine regulations permit.

